# Predicting survival in patients with buccal cancer: A study based on SEER database and external validation in China

**DOI:** 10.1002/cam4.6907

**Published:** 2024-01-29

**Authors:** Yongmei Tan, Guoxing Huang, Jintao Hu, Shaoping Zhao, Yanyan Li, Zhihui Wen, Liansheng Wang, Suling Chen, Rongxi Chen, Haotian Cao, Jinsong Li

**Affiliations:** ^1^ Department of Oral and Maxillofacial Surgery, Department of General Dentistry, Sun Yat‐sen Memorial Hospital Sun Yat‐sen University Guangzhou Guangdong P. R. China; ^2^ State Key Laboratory of Oncology in South China, Collaborative Innovation Center for Cancer Medicine Sun Yat‐sen University Cancer Center Guangzhou Guangdong P. R. China; ^3^ Department of Urology, Sun Yat‐sen Memorial Hospital Sun Yat‐sen University Guangzhou Guangdong P. R. China; ^4^ Department of Stomatology Guangzhou Baiyun District Maternal and Child Health Hospital Guangzhou Guangdong P. R. China

**Keywords:** buccal mucosa cancer, cancer‐specific survival, nomogram, overall survival, prognosis

## Abstract

**Objective:**

Buccal mucosa cancer (BMC) is one of the most common oral cancers and has poor prognosis. The study aimed to develop and validate nomograms for predicting the 1‐, 3‐, and 5‐year overall survival (OS) and cancer‐specific survival (CSS) of BMC patients.

**Methods:**

We collected and reviewed information on BMC patients diagnosed between 2004 and 2019 from the Surveillance Epidemiology and End Results database. Two nomograms were developed and validated to predict the OS and CSS based on predictors identified by univariate and multivariate Cox regression. An extra external validation was further performed using data from Sun Yat‐sen Memorial Hospital (SYSMH).

**Results:**

A total of 3154 BMC patients included in this study were randomly assigned to training and validation groups in a 2:1 ratio. Independent prognostic predictors were identified, confirmed, and fitted into nomograms for OS and CSS, respectively. The C‐indices are 0.767 (Training group OS), 0.801 (Training group CSS), 0.763 (Validation group OS), and 0.781 (Validation group OS), respectively. Moreover, the nomograms exhibited remarkable precision in forecasting and significant clinical significance, as evidenced by receiver operating characteristic (ROC) curves, calibration curves, and decision curve analyses (DCA). The final validation using our data from SYSMH also showed high accuracy and substantial clinical benefits within the nomograms. The C‐indices are 0.849 (SYSMH group OS) and 0.916 (SYSMH group CSS). These indexes are better than tumor, node, and metastasis stage based on prediction results.

**Conclusions:**

The nomograms developed with great performance predicted 1‐, 3‐, and 5‐year OS and CSS of BMC patients. Use of the nomograms in clinical practices shall bring significant benefits to BMC patients.

## 1 INTRODUCTION

Buccal mucosa cancer (BMC) is a type of oral cancer commonly found in low‐ and middle‐income countries in South Central Asia, as well as Melanesia, which has been associated with the popularity of betel nut and tobacco chewing.^1^, ^2^ BMC exhibits a strong propensity for aggressiveness, with a stage III or IV tumor present in 34% of patients and over 60% of patients experiencing relapse within the initial 2 years following diagnosis.^3^ Currently, the main treatments for BMC are surgery plus chemo‐ and/or radiotherapy, in addition to immunotherapy, which may be a better option for some of the patients.^4^, ^5^, ^6^ Studies have also compared the differences in the failure pattern of buccal and other types of oral cancer and have suggested that treatments may be adapted to patients' risk by stratification via prognostic models for these malignancies.^7^, ^8^ Identifying and evaluating risk factors, in turn, predicting the prognosis of BMC to inform clinical decisions has become increasingly important for BMC treatment and care.

The current clinical practice relies on the AJCC staging system to predict the prognosis of BMC, which has been predominantly based on the tumor, node, and metastasis (TNM) staging of tumors. The recently published eighth edition has evolved and further included the depth of invasion, extranodal extension, and HPV infection as determinants of prognosis.^9^ However, the prognostic efficacy and reliability of this staging system are still being questioned due to its relatively low‐predictive power because it fails to take into consideration other important prognostic factors. For example, studies have demonstrated that age, histologic subtypes, surgical therapies, and even race and sex may have influences on the prognosis of BMC.^10^, ^11^ Thus, considering the crucial role of other prognostic factors and the limitations of AJCC staging in the prediction of BMC patients, it is necessary to develop a more accurate predictive system considering more prognostic predictors of BMC.

A nomogram is an intuitive scoring system that predicts the cumulative effects of each variable on a specific outcome by assigning each value of the included variables a point based on their contribution to the outcome.^12^ In comparison with the conventional AJCC staging system, nomograms could encompass substantially more prognostic predictors, including some sociodemographic and clinicopathologic parameters, to predict long‐term survival.^12^, ^13^, ^14^ The clinical applicability of nomograms in predicting prognosis has been suggested in many malignancies, including cardiac tumors, endometrial carcinoma, and medulloblastoma.^15^, ^16^, ^17^ Moreover, the applicability of nomograms in predicting BMC prognosis has gained increasing attention recently, and some nomograms have already been developed.^10^, ^11^, ^18^, ^19^ However, the factors identified by the previous studies have been varying, even some of the studies were based on the same database. More importantly, no study has ever validated the performance of nomograms in predicting BMC prognosis by an extra dataset beyond the original dataset.

In the present study, we seek to develop nomograms for and validate their performance in predicting 1‐, 3‐, and 5‐year OS and CSS of BMC patients. Furthermore, we will, for the first time per our knowledge, assess and validate the performance of nomograms in predicting the prognosis of Asian BMC patients by using data archived in our hospital.

## 2 MATERIALS AND METHODS

### 2.1 Data source

In the present investigation, the current study utilized the Surveillance Epidemiology and End Results (SEER) database, supported by the Surveillance Research Program (SRP) in NCI's Division of Cancer Control and Population Sciences (DCCPS). All authors have gained access to the data as required by the database. Data extraction was performed using SEER*Stat software Version 8.4.1.

### 2.2 Patients and covariates

**FIGURE 1:**
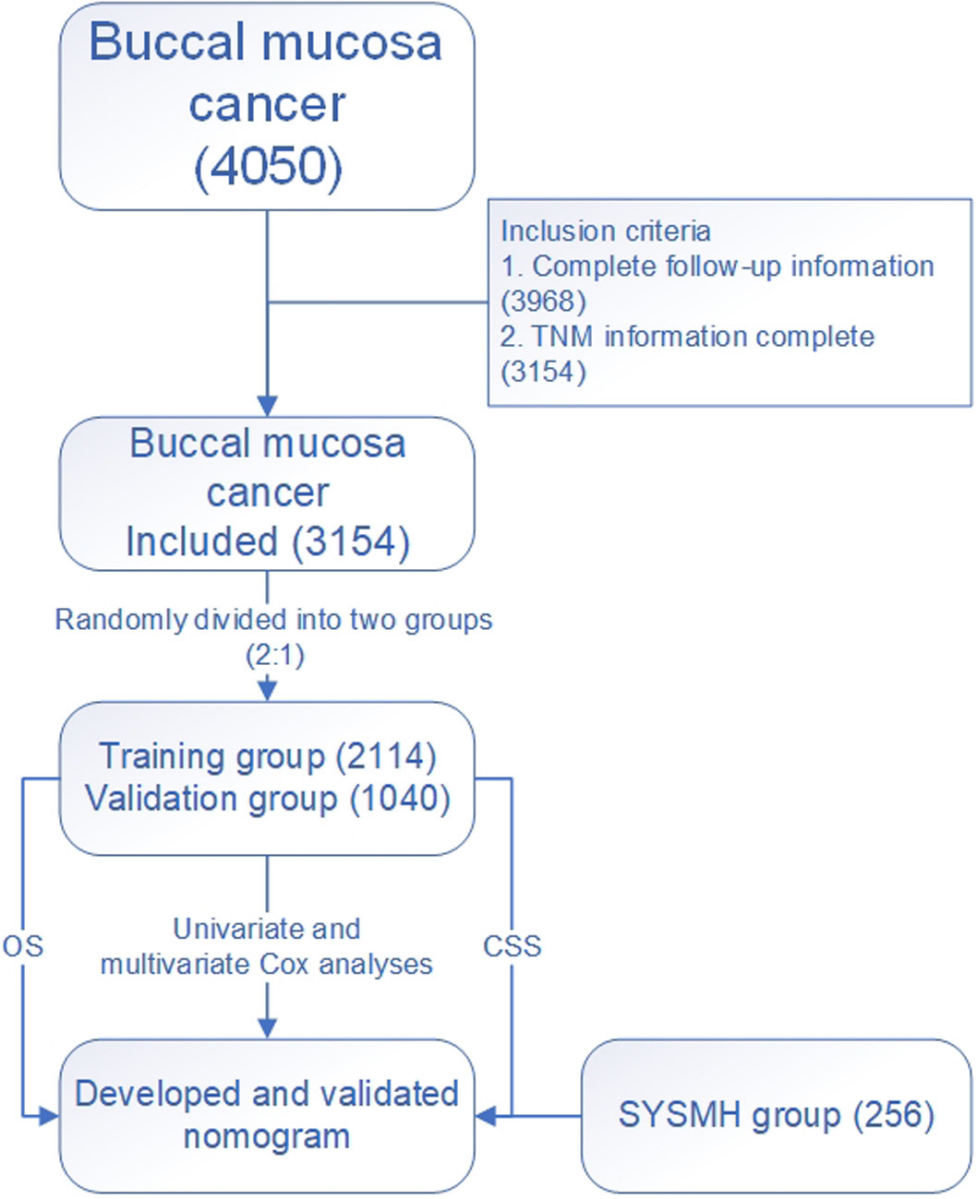
The flow chart of this investigation.

We identified and retrospectively analyzed BMC patients diagnosed between 2004 and 2019 from the SEER database. The data collected encompassed demographics (including sex, age, race, marital status, income, and living area), TNM staging, tumor histology and grade, primary tumor location and tumor number, treatment characteristics (including surgery, radiation, and chemotherapy), and survival outcomes of patients. Patients with incomplete information on follow‐up or TNM staging were excluded from analysis in the current study. Afterward, patients included in the study were randomly assigned to training and validation groups in a 2:1 ratio. The training cohort was used to develop nomograms for the prediction of OS and CSS, of which the predictive performances were further assessed and validated using both the training cohort (internal) and the validation cohort (external). A flow chart of the selection process in the current study is presented in Figure 1. In addition, an extra external validation of the nomograms was conducted using historical data archived at Sun Yat‐sen Memorial Hospital (SYSMH). Our Institutional Review Board approved the study, which included 256 patients with BMC who were diagnosed at the hospital and then underwent surgery between 2004 and 2019.

### 2.3 Development of nomograms for prediction of OS and CSS of BMC patients

To develop nomograms for predicting OS and CSS of BMC patients, a univariate Cox proportional hazards regression analysis on the training data was first used to screen factors that might significantly impact the OS and CSS, respectively, followed by a multivariate Cox regression analysis to confirm the independence of those screened risk factors. Variables with a *p* < 0.05 in the univariate Cox regression analysis were recruited in the multivariate Cox regression analysis in a forward‐stepwise manner and were removed if they failed to meet the likelihood ratio criterion. The corresponding hazard ratios (HR) and 95% confidence intervals (CI) were also calculated for each included variable in both univariate and multivariate regression analyses. Based on the selected variables and the corresponding survival data in the training cohort, prognostic nomograms were fitted for the prediction of 1‐, 3‐, and 5‐year OS and CSS, respectively.

### 2.4 Assessment and validation of nomograms for prediction of OS and CSS

To estimate a patient's OS and CSS probabilities over a specified duration, follow these steps: Firstly, ascertain the corresponding points for each variable. Then, calculate the total points by summing the individual scores of these variables. The probability associated with this total score represents the predicted outcome. To further assess and validate the predictive performance of the nomograms in terms of discrimination, concordance, and clinical applicability, ROC curves, calibration curves, and DCA curves were used, respectively. Furthermore, the prediction efficiency of Nomogram model was compared with TNM staging.

### 2.5 Statistical analysis

Patients' demographic and clinicopathological data were presented as *n* (%). The survival curves of OS and CSS were compared by Kaplan–Meier survival analysis with GraphPad Prism 7.0 (GraphPad Software, Boston, MA). Statistical analyses for univariate and multivariate Cox regression were conducted using SPSS 26.0 (IBM SPSS Statistics, Armonk, NY). The nomograms were plotted and validated by executing the *rms*, *hmisc*, *survival*, *formula*, *ggplot2*, and *timeROC* packages in R Statistical Software (v4.2.1; R Core Team 2021). In the current investigation, a significance level of less than 0.05 was deemed statistically significant.

## 3 RESULTS

### 3.1 Baseline demographic and clinical characteristics

A total of 4050 BMC patients were identified from the SEER database, from which 3154 patients, based on our criteria, were included in the current study, with 2114 and 1040 patients assigned to the training and validation group, respectively. As shown in Table 1, the vast majority of the patients were White (78.31%) with an age older than 58 years (73.87%) and lived in a rural area (86.05%) with an annual household income of over $60,000 (67.85%). The primary tumors were located predominantly on cheek mucosa (93.69%), of which the most prevalent type was squamous cell carcinoma (83.23%). Regarding tumor grade, moderately differentiated tumors accounted for 45.15% of all the tumors, followed by well‐differentiated (28.22%) and poorly differentiated (13.06%). As for the TNM staging, most patients were in the M0 stage (98.29%), while the distribution across the T staging scheme was relatively even. Moreover, most of the tumors were in the N0 stage (71.12%), followed by the N2c stage (12.56%) and N1 stage (11.57%). Regarding the treatments, most of the patients underwent various degrees of surgery, including radical excision of tumor (32.24%), wide excision (29.55%), and local tumor excision (22.76%). In addition, a small part of the patients was clearly documented to have chemotherapy (19.12%), whereas nearly half of the patients underwent radiotherapy (42.14%).

**TABLE 1:** Baseline characteristics of patients included.

	SEER		SYSMH	
	No.	%	No.	%
Overall	3154		256	
Sex				
Male	1722	54.60	139	54.30
Female	1432	45.40	117	45.70
Age				
<45	247	7.83	27	10.55
46–57	577	18.29	163	63.67
>58	2330	73.87	66	25.78
Race				
White	2470	78.31	0	0.00
Black	213	6.75	0	0.00
Other	471	14.93	256	100.00
Primary tumor location				
Cheek mucosa	2955	93.69	242	94.53
Vestibule of mouth	199	6.31	14	5.47
Histology				
Squamous cell carcinoma	2625	83.23	248	96.88
Other/Unknown	529	16.77	8	3.13
Grade				
Well differentiated	890	28.22	61	23.83
Moderately differentiated	1424	45.15	103	41.02
Poorly differentiated	412	13.06	16	6.25
Undifferentiated	15	0.48	2	0.00
Unknown	413	13.09	74	28.91
T stage				
T1	1256	39.82	52	20.31
T2	928	29.42	124	48.44
T3	332	10.53	29	11.33
T4a	538	17.06	40	15.63
T4b	100	3.17	11	4.30
N stage				
N0	2243	71.12	148	57.81
N1	365	11.57	53	20.70
N2a	63	2.00	2	0.78
N2b	43	1.36	38	14.84
N2c	396	12.56	7	2.73
N3	44	1.40	8	3.13
M stage				
M0	3100	98.29	253	98.83
M1	54	1.71	3	1.17
Only one primary				
No	1178	37.35	242	94.53
Yes	1976	62.65	14	5.47
Surgery				
No surgery performed	463	14.68	40	15.63
Local tumor excision	718	22.76	17	6.64
Wide excision	932	29.55	95	37.11
Radical excision of tumor	1017	32.24	99	38.67
Unknown	24	0.76	5	1.95
Radiation				
No/Unknown	1825	57.86	241	94.14
Yes	1329	42.14	15	5.86
Chemotherapy				
No/Unknown	2551	80.88	247	96.48
Yes	603	19.12	9	3.52
Marital				
Married	1699	53.87	237	92.58
Never married/Single	456	14.46	4	1.56
Separated/Divorced	253	8.02	4	1.56
Widowed	509	16.14	7	2.73
Other/Unknown	237	7.51	4	1.56
Income				
<$6000	1014	32.15	–	–
>$6000	2140	67.85	–	–
Rural–Urban				
Rural	2714	86.05	142	55.47
Urban	440	13.95	114	44.53
Status				
Alive	1609	51.01	200	78.13
Dead	1545	48.99	56	21.88

The external validation cohort comprised 256 Asian patients that were treated in our institute between 2010 and 2021. Compared to the SEER dataset, except for a similar male/female ratio, several profound differences in patient characteristics were noticed: first, the patients were younger (63.67% of the patients were 46–57 years old), and nearly half of them lived in the urban area (44.53% vs. 13.95%). Second, a relatively higher proportion of tumors with unknown grades (28.91% vs. 13.09%) and N2b stage (14.84% vs. 1.36%) were reported, and most of the patients carried more than one primary tumor (94.53% vs. 37.35%). Lastly, patients in our hospital tended to undergo wide and radical excision than local excision, and patients with a clearly recorded history of radiation therapy were significantly less (5.86% vs. 42.14%).

### 3.2 Factors associated with OS and CSS

**FIGURE 2:**
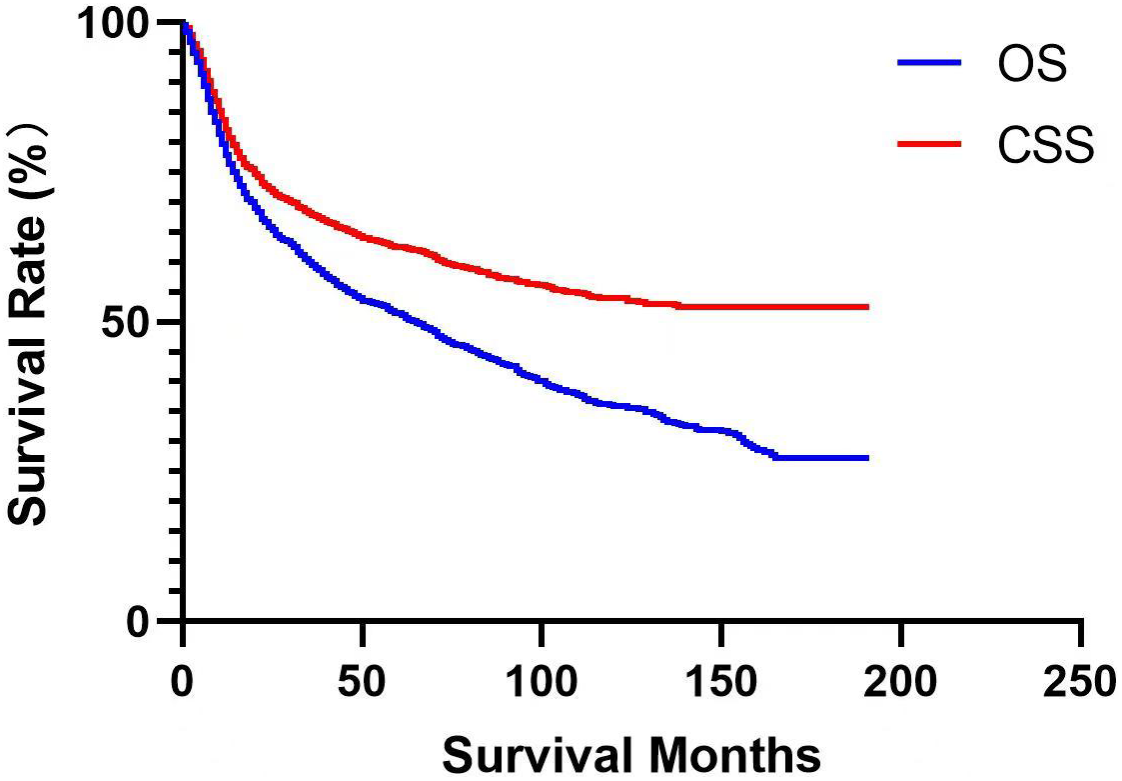
Kaplan–Meier analysis and log‐rank test were utilized to examine the overall survival (OS) and cancer‐specific survival (CSS) of the buccal mucosa cancer patients from Surveillance Epidemiology and End Results.

As of the date of data extraction, 51.01% of the patients enrolled in the SEER dataset were still alive (Table 1). The 1‐, 3‐, and 5‐year OS rates for patients with BMC were 77.88%, 59.49%, and 51.45%, respectively, while the 1‐, 3‐, and 5‐year CSS rates were 82.08%, 67.78%, and 62.57%, respectively (Figure 2). Overall, the median survival of BMC patients was 65 months. However, the median CSS could not be defined in the current study because the cancer‐specific death rate did not reached 50% yet by the time of the study (Figure 2). The screening for independent prognostic variables by univariate Cox regression analysis demonstrated that age, race/ethnicity, histology, grade, TNM stage, surgery, radiotherapy, chemotherapy, marital status, income, and living areas may have significant influences on both the OS and CSS, in addition to the number of primary tumors which may impact the OS only (Tables 2 and 3). Further multivariate Cox regression analysis confirmed that those variables screened by univariate analysis were all independent risk factors for OS, except for race/ethnicity and income (Table 2). Furthermore, chemotherapy, in addition to race/ethnicity and income, was also excluded by multivariate Cox regression analysis for CSS (Table 3).

**TABLE 2:** Univariate and multivariate Cox analyses of patients included (OS).

Variable	Univariate analysis			Multivariate analysis (OS)		
	HR	95% CI	*p* value	HR	95% CI	*p* value
Sex			0.682			
Male	1 (ref)					
Female	0.979	0.886–1.082	0.682			
Age			<0.001			<0.001
<45	1 (ref)			1 (ref)		
46–57	1.339	1.017–1.762	0.037	1.246	0.944–1.645	0.121
>58	2.807	2.205–3.574	<0.001	2.325	1.809–2.989	<0.001
Race			0.001			
White	1 (ref)					
Black	0.953	0.783–1.16	0.631			
Other	0.749	0.641–0.874	<0.001			
Primary tumor location			0.143			
Cheek mucosa	1 (ref)					
Vestibule of mouth	0.859	0.698–1.057	0.152			
Histology			<0.001			<0.001
Squamous cell carcinoma	1 (ref)			1 (ref)		
Other/Unknown	0.346	0.29–0.411	<0.001	0.528	0.437–0.637	
Grade			<0.001			<0.001
Well differentiated	1 (ref)			1 (ref)		
Moderately differentiated	1.426	1.253–1.622	<0.001	1.242	1.087–1.419	0.001
Poorly differentiated	2.233	1.902–2.62	<0.001	1.73	1.46–2.049	<0.001
Undifferentiated	2.589	1.42–4.72	0.002	4.054	2.2–7.469	<0.001
Unknown	1.320	1.111–1.569	0.002	1.186	0.988–1.423	0.067
T stage			<0.001			<0.001
T1	1 (ref)			1 (ref)		
T2	1.752	1.541–1.992	<0.001	1.42	1.239–1.627	<0.001
T3	2.768	2.346–3.265	<0.001	2.084	1.737–2.499	<0.001
T4a	3.300	2.862–3.804	<0.001	2.205	1.864–2.61	<0.001
T4b	4.131	3.187–5.356	<0.001	1.67	1.25–2.23	0.001
N stage			<0.001			<0.001
N0	1 (ref)			1 (ref)		
N1	0.351	0.219–0.562	<0.001	1.74	1.493–2.028	<0.001
N2a	0.746	0.461–1.209	0.234	2.063	1.381–3.08	<0.001
N2b	0.921	0.504–1.682	0.788	2.645	2.27–3.082	<0.001
N2c	1.060	0.657–1.711	0.810	3.029	2.123–4.322	<0.001
N3	1.788	1.015–3.152	0.044	2.617	1.616–4.238	<0.001
M stage			<0.001			<0.001
M0	1 (ref)			1 (ref)		
M1	4.637	3.484–6.172	<0.001	2.011	1.484–2.725	
Only one primary			<0.001			0.036
No	1 (ref)		0.001	1 (ref)		
Yes	0.842	0.761–0.931		0.894	0.804–0.993	
Surgery			<0.001			<0.001
No surgery performed	1 (ref)			1 (ref)		
Local tumor excision	0.243	0.209–0.283	<0.001	0.396	0.332–0.472	<0.001
Wide excision	0.218	0.188–0.252	<0.001	0.336	0.284–0.398	<0.001
Radical excision of tumor	0.331	0.289–0.379	<0.001	0.311	0.267–0.362	<0.001
Unknown	0.629	0.387–1.023	0.062	0.494	0.301–0.812	0.005
Radiation			<0.001			<0.001
No/Unknown	1 (ref)			1 (ref)		
Yes	1.440	1.303–1.592	<0.001	0.732	0.65–0.825	
Chemotherapy			<0.001			0.046
No/Unknown	1 (ref)			1 (ref)		
Yes	1.839	1.637–2.066	<0.001	0.864	0.749–0.997	
Marital						<0.001
Married	1 (ref)			1 (ref)		
Never married/Single	1.112	0.956–1.293	0.170	1.152	0.985–1.346	0.076
Separated/Divorced	1.269	1.054–1.527	0.012	1.086	0.9–1.311	0.387
Widowed	1.969	1.733–2.238	0.000	1.34	1.172–1.532	<0.001
Other/Unknown	0.783	0.628–0.976	0.030	0.667	0.532–0.835	<0.001
Income			0.001			
<$6000	1 (ref)					
>$6000	0.841	0.757–0.934	0.001			
Rural–Urban			0.001			0.005
Rural	1 (ref)			1 (ref)		
Urban	0.789	0.689–0.904	0.001	0.819	0.713–0.94	

**TABLE 3:** Univariate and multivariate Cox analyses of patients included (CSS).

Variable	Univariate analysis			Multivariate analysis (CSS)		
	HR	95% CI	*p* value	HR	95% CI	*p* value
Sex			0.974306			
Male	1 (ref)					
Female	0.998	0.884–1.127	0.974306			
Age			<0.001			<0.001
<45	1 (ref)			1 (ref)		
46 – 57	1.160	0.865–1.557	0.321	1.092	0.81–1.473	0.563
>58	1.852	1.433–2.394	<0.001	1.681	1.286–2.198	<0.001
Race			0.033576			
White	1 (ref)					
Black	1.001	0.792–1.266	0.991			
Other	0.789	0.656–0.949	0.012			
Primary tumor location			0.135			
Cheek mucosa	1 (ref)					
Vestibule of mouth	0.825	0.637–1.069	0.14583			
Histology			<0.001			<0.001
Squamous cell carcinoma	1 (ref)			1 (ref)		
Other/Unknown	0.282	0.223–0.358	<0.001	0.459	0.356–0.592	
Grade			<0.001			<0.001
Well differentiated	1 (ref)			1 (ref)		
Moderately differentiated	1.563	1.332–1.835	<0.001	1.252	1.061–1.478	0.008
Poorly differentiated	2.655	2.194–3.213	<0.001	1.902	1.554–2.328	<0.001
Undifferentiated	3.499	1.856–6.599	<0.001	6.124	3.19–11.757	<0.001
Unknown	1.234	0.986–1.543	0.066	1.038	0.821–1.313	0.753
T stage			<0.001			<0.001
T1	1 (ref)			1 (ref)		
T2	1.919	1.629–2.261	<0.001	1.447	1.217–1.721	<0.001
T3	3.411	2.793–4.166	<0.001	2.269	1.821–2.827	<0.001
T4a	4.173	3.515–4.954	<0.001	2.362	1.928–2.894	<0.001
T4b	5.484	4.105–7.327	<0.001	1.737	1.257–2.4	0.001
N stage			<0.001			<0.001
N0	1 (ref)			1 (ref)		
N1	2.682	2.267–3.172	<0.001	2.009	1.683–2.399	<0.001
N2a	3.763	2.478–5.713	<0.001	2.604	1.694–4.003	<0.001
N2b	4.142	3.56–4.82	<0.001	3.085	2.604–3.655	<0.001
N2c	7.443	5.23–10.594	<0.001	3.403	2.326–4.98	<0.001
N3	3.554	2.118–5.962	<0.001	2.789	1.643–4.737	<0.001
M stage			<0.001			<0.001
M0	1 (ref)			1 (ref)		
M1	5.705	4.198–7.753	<0.001	2.286	1.644–3.178	
Only one primary			0.597			
No	1 (ref)					
Yes	1.034	0.913–1.171	0.598			<0.001
Surgery			<0.001			
No surgery performed	1 (ref)			1 (ref)		
Local tumor excision	0.196	0.163–0.237	<0.001	0.367	0.296–0.456	<0.001
Wide excision	0.191	0.16–0.228	<0.001	0.323	0.264–0.395	<0.001
Radical excision of tumor	0.328	0.28–0.384	<0.001	0.298	0.251–0.355	<0.001
Unknown	0.587	0.33–1.046	0.071	0.474	0.264–0.852	0.012
Radiation			<0.001			<0.001
No/Unknown	1 (ref)			1 (ref)		
Yes	1.692	1.498–1.91	<0.001	0.723	0.63–0.83	
Chemotherapy			<0.001			
No/Unknown	1 (ref)					
Yes	2.363	2.071–2.697	<0.001			
Marital			<0.001			<0.001
Married	1 (ref)			1 (ref)		
Never married/Single	1.278	1.073–1.521	0.006	1.248	1.042–1.495	0.016
Separated/Divorced	1.267	1.014–1.583	0.037	1.062	0.847–1.332	0.600
Widowed	1.774	1.514–2.079	0	1.288	1.091–1.52	0.003
Other/Unknown	0.703	0.53–0.932	0.014	0.622	0.467–0.829	0.001
Income			0.012			
<$6000	1 (ref)					
>$6000	0.849	0.748–0.964	0.011			
Rural–Urban			0.009			0.006
Rural	1 (ref)			1 (ref)		
Urban	0.798	0.676–0.941	0.007	0.791	0.669–0.935	

### 3.3 Nomograms for prediction of OS and CSS of BMC patients

**FIGURE 3:**
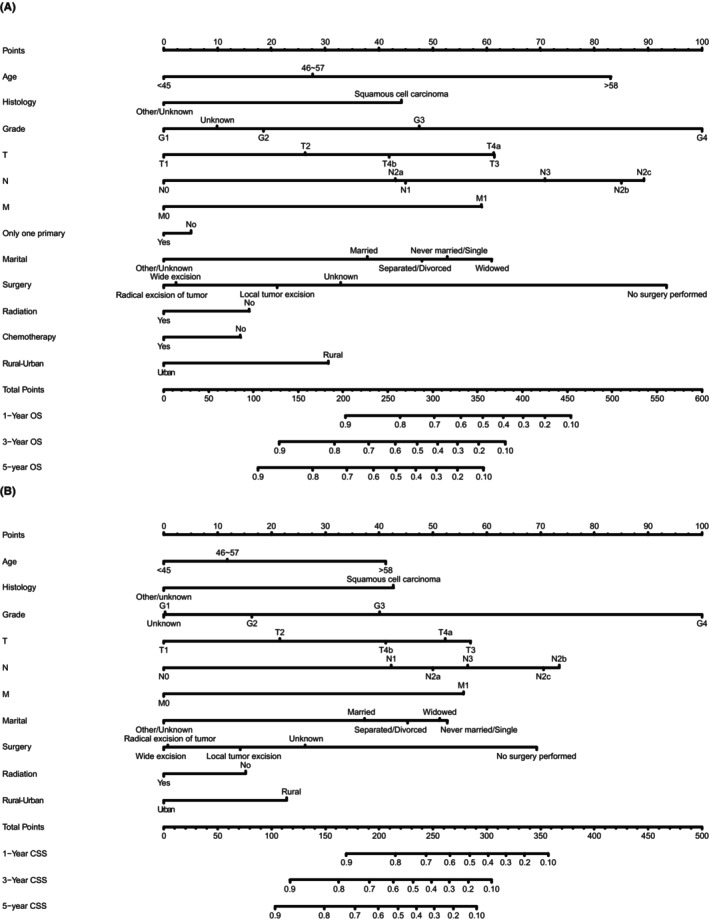
Nomogram predicting 1‐, 3‐, and 5‐year overall survival (OS) (A) and cancer‐specific survival (CSS) (B) for patients with buccal mucosa cancer.

Based on the results of the multivariate Cox regression analysis, age, histology, grade, TNM stage, primary tumor number, surgery, radiotherapy, chemotherapy, marital status, and living area were fitted into the nomogram for OS, while primary tumor number and chemotherapy were excluded from the nomogram for CSS (Figure 3). From the nomograms depicted in Figure 3, a general interpretation of the contribution of various variables to the survival of the patients could be drawn. For example, tumor grade contributed the most to both OS and CSS, followed by surgery and N staging. On the other end, primary tumor number and radiotherapy contributed the least to OS and CSS, respectively (Figure 3).

### 3.4 Assessment and validation of nomograms

**FIGURE 4:**
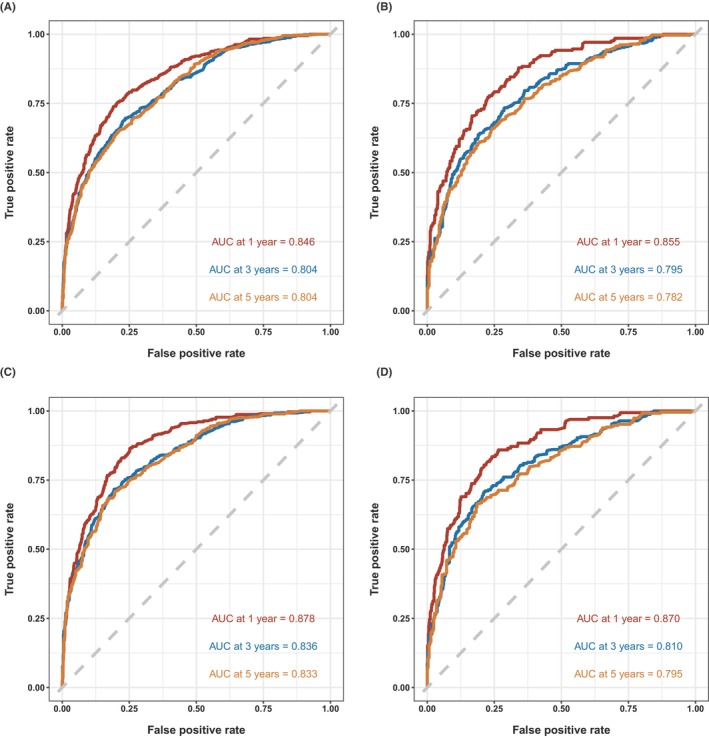
Receiver operating characteristic analysis for evaluating the accuracy for 1‐, 3‐, and 5‐year overall survival (OS) and cancer‐specific survival (CSS) nomogram. (A, B) OS and CSS of the training group and (C, D) OS and CSS of the validation group.

**FIGURE 5:**
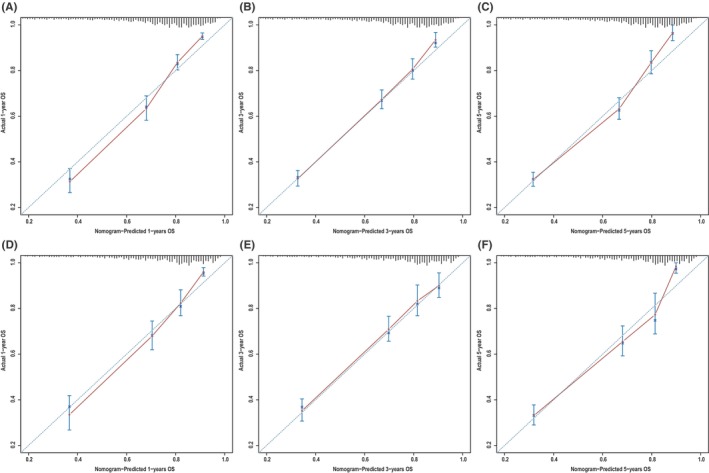
On the x‐axis, the predicted overall survival (OS) is represented by nomogram A, while the actual OS is represented on the y‐axis. (A–C) Training group and (D–F) validation group.

**FIGURE 6:**
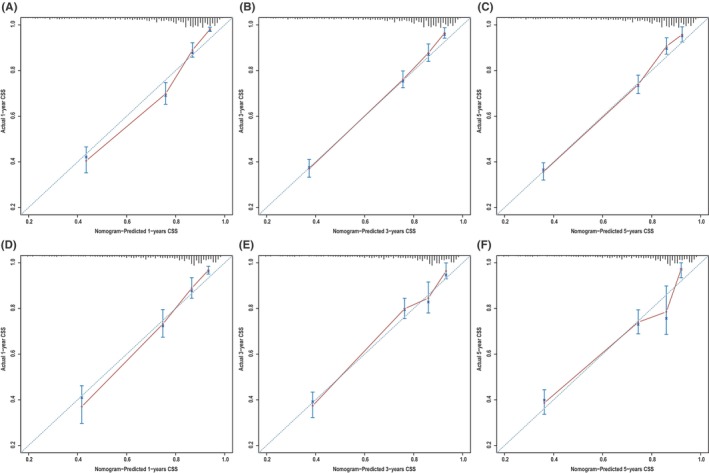
Calibration plots of nomogram B for predicting 1‐, 3‐, and 5‐year cancer‐specific survival (CSS). Nomogram‐predicted CSS is plotted on the x‐axis; actual CSS is plotted on the y‐axis. (A–C) Training group and (D–F) validation group.

**FIGURE 7:**
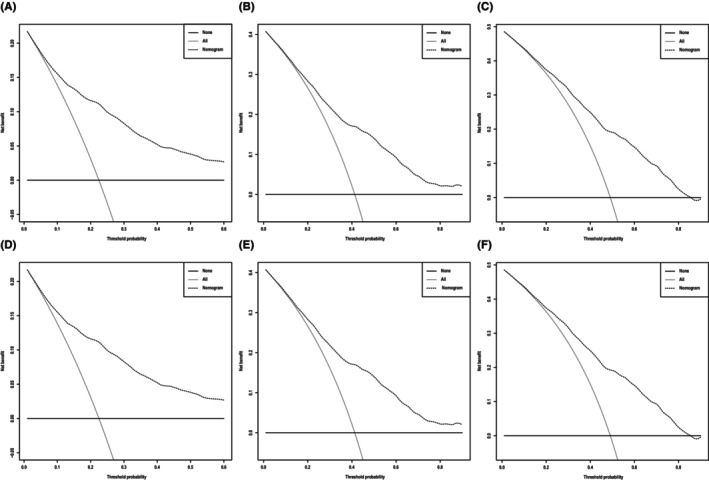
Decision curve analysis, which assesses the net benefit of the overall survival (OS) nomogram. (A–C) Net benefit of OS at 1, 3, and 5 years in the training group and (D–F) net benefit of OS at 1, 3, and 5 years in the validation group.

**FIGURE 8:**
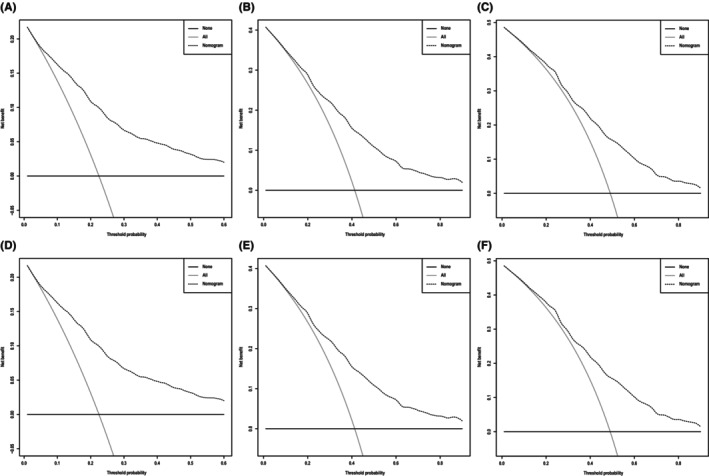
Decision curve analysis, which assesses the net benefit of the cancer‐specific survival (CSS) nomogram. (A–C) Net benefit of CSS at 1, 3, and 5 years in the training group and (D–F) net benefit of CSS at 1, 3, and 5 years in the validation group.

In the current study, we divided the SEER data into a training cohort and a validation cohort in a ratio of 2:1. Upon the development of the nomograms, we not only used an internal validation by bootstrap resampling method but also a cohort of external validation to assess and validate the performance of the nomograms. The C‐indices are 0.767 (Training group OS), 0.801 (Training group CSS), 0.763 (Validation group OS), and 0.781 (Validation group OS), respectively. Figure 4 illustrates the ROC curves depicting the discrimination capabilities of the nomograms for OS and CSS, using data from the training and validation cohorts, respectively. Generally, the OS and CSS nomograms demonstrated medium power to accurately classify survival, which decreased with the predicted time increased from 1 to 5 years. For the OS nomogram, the area under ROC curves (AUC) for 1‐, 3‐, and 5‐year predictions using the training cohort were 0.846, 0.804, and 0.804, respectively (Figure 4A **)**, which corresponded to AUCs of 0.855, 0.795, and 0.782 using the validation cohort, respectively (Figure 4B). Similarly, the AUCs of ROC curves for 1‐, 3‐, and 5‐year CSS predictions of the training cohort were 0.878, 0.836, and 0.833, respectively (Figure 4C), while the AUCs of 1‐, 3‐, and 5‐year CSS prediction using the validation cohort were 0.870, 0.810, and 0.795, respectively (Figure 4D). In addition to the ROC curves, we also used calibration curves to assess the consistency between the predicted and actual 1‐, 3‐, and 5‐year OS and CSS, respectively. As shown in Figures 5 and 6, the calibration curves for 1‐, 3‐, and 5‐year predictions of OS in the training cohort and the validation cohort aligned well with the corresponding reference lines (Figure 5), which was the same case for CSS predictions (Figure 6), it is evident that the predicted and actual survival rates are highly consistent both in the training cohort and in the validation cohort. Furthermore, the higher net benefit of the DCA analyses demonstrated that using the nomograms for the prediction of OS (Figure 7) and CSS (Figure 8) to inform clinical decisions on patients in both the training cohort and the validation cohort will lead to superior outcomes for any decision associated with a threshold probability of above 20% or so.

**FIGURE 9:**
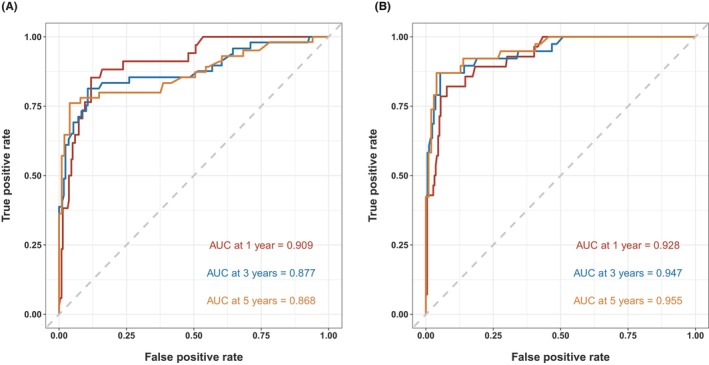
Receiver operating characteristic analysis for evaluating the accuracy for 1‐, 3‐, and 5‐year overall survival (OS) and cancer‐specific survival (CSS) nomogram. (A) OS of the Sun Yat‐sen Memorial Hospital group and (B) CSS of the SYSMH group.

**FIGURE 10:**
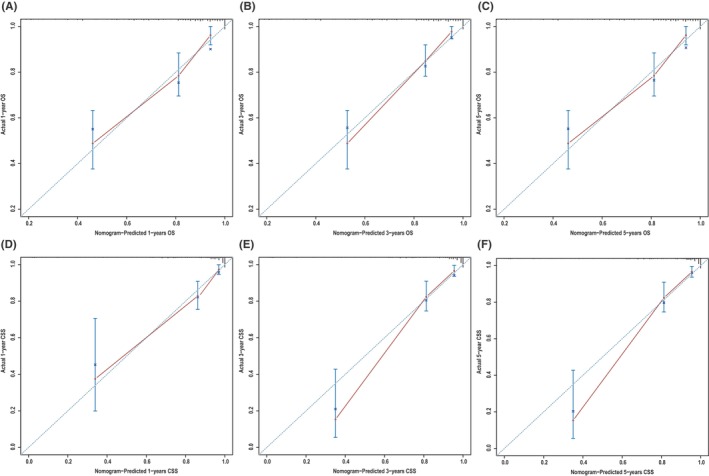
Calibration plots of nomogram A for predicting 1‐, 3‐, and 5‐year overall survival (OS) and cancer‐specific survival (CSS). (A–C) OS of Sun Yat‐sen Memorial Hospital (SYSMH) group and (D–F) CSS of SYSMH group.

**FIGURE 11:**
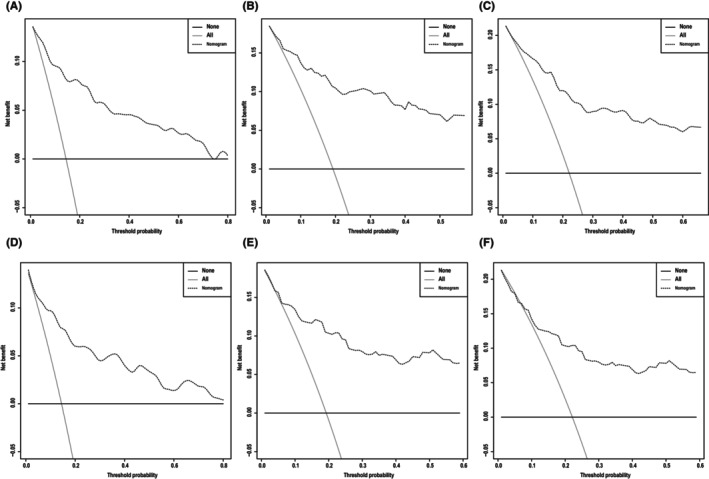
Decision curve analysis, which assesses the net benefit of the overall survival (OS) and cancer‐specific survival (CSS) nomogram. (A–C) Net benefit of OS at 1, 3, and 5 years in the SYSMH group and (D–F) net benefit of CSS at 1, 3, and 5 years in the SYSMH group.

In addition to the validation cohort from the SEER database, we further validated the practical application of the nomograms by using archived data of patients treated in our hospital. The C‐indices are 0.849 (SYSMH group OS) and 0.916 (SYSMH group CSS). As shown in Figure 9, the nomograms demonstrated a high power of accuracy in predicting the OS and CSS of BMC patients in our hospital. The AUCs of ROC curves for 1‐, 3‐, and 5‐year OS prediction reached 0.909, 0.877, and 0.868, respectively, while the AUCs of ROC curves for 1‐, 3‐, and 5‐year CSS prediction reached 0.928, 0.947, and 0.955, respectively (Figure 9). The calibration curves also demonstrated high consistency between the predicted and actual survival of patients in terms of both OS and CSS (Figure 10). Lastly, the DCA curves indicated even larger net benefits could be obtained from clinical decisions based on the predictions for OS and CSS compared to that of the patients in the SEER database (Figure 11).

**FIGURE 12:**
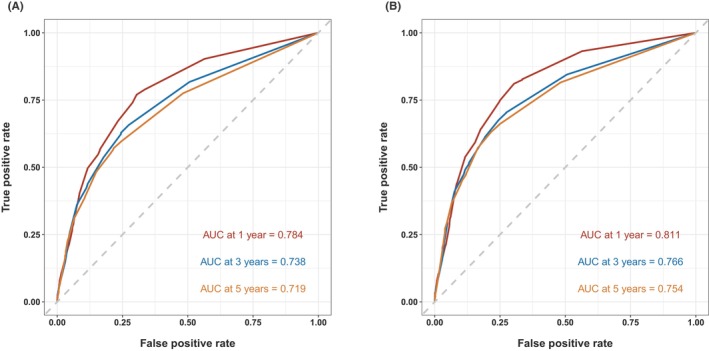
Receiver operating characteristic analysis to assess the accuracy of predicting 1‐, 3‐, and 5‐year overall survival (A) and cancer‐specific survival (B) in buccal mucosa cancer patients based on the AJCC TNM staging.

The C‐index for predicting OS and CSS based on the AJCC TNM staging was 0.693 and 0.731, respectively, with ROC curves presented in Figure 12. Our nomogram demonstrated superior predictive capabilities compared to the TNM staging.

## 4 DISCUSSION

In the present study, we developed two nomograms to predict the 1‐, 3‐, and 5‐year OS and CSS of BMC patients, respectively, using data on survival and covariates from the openly accessible SEER cancer registry. In addition, we validated the performance and clinical applicability of the nomograms by not only an external dataset generated from the same database but also an extra external dataset from our hospital. The evaluation and confirmation of the nomograms through ROC curves, calibration curves, and DCA demonstrate that the nomograms exhibit excellent predictive ability in terms of differentiation, agreement, and practicality in clinical settings.

Currently, the clinical practice of predicting the prognosis of BMC patients predominantly relies on the AJCC staging system, which mostly depends on the TNM staging of tumors. Consistent with the AJCC staging, our study demonstrated that the TNM staging was not only an independent predictor but also contributed significantly to the survival of both the OS and CSS of BMC patients. However, previous studies have clearly indicated that significant differences in individual survival may present even if patients were classified into the same TNM stage.^19^ Thus, the AJCC staging system has been criticized for inherent defects within its prediction using only TNM staging, and more prognostic factors should be included to more accurately predict the survival of cancer patients. In our developed nomograms for predicting OS and CSS, the differentiation grade of the tumor showed more pronounced influences on the survival of BMC patients than TNM staging did. This is consistent with previous studies, which demonstrated that in surgically treated T4 stage BMC, the tumor differentiation contributed the most to patient survival, superior to lymph node involvement, perineural invasion, and bone infiltration.^19^ In addition, our model demonstrates that treatment strategies, including surgery, radiation, and chemotherapy, could all significantly improve the OS of BMC patients, indicating that surgery plus adjuvant therapies may improve the survival of BMC patients even longer. Indeed, Comer et al. recently reported that BMC patients with lymphovascular invasion treated with surgery plus postoperative radiotherapy or postoperative chemoradiotherapy had significantly improved survival compared to those who were treated with surgery alone.^20^ Our nomogram for OS also indicates that the number of primary tumors could have significant impacts on patient survival, which has also been proven by previous studies demonstrating that multiple primary tumors could significantly reduce the survival of patients with oral cancer.^21^, ^22^ Previous studies have also demonstrated that squamous cell carcinoma of the maxillary sinus has worse survival than non‐squamous malignancies.^23^ And we, for the first time per our best knowledge, identified that this is also true for the histological type of BMC, though it was only significant for OS. Lastly, demographic characteristics like age, marital status, and living area of patients were also found to be related to patient survival. In regard to age, our models showed that patients older than 79 might have a drastically worst prognosis than patients younger than 78. In comparison with the survival data of patients in our hospital, patients in the SEER database were older and had worst survival. Life events like the loss of a partner may significantly decrease survival, whereas living in a rural area could significantly improve patient survival. Together, the nomograms developed in the current study could be used not only by the clinicians to guide their clinical practices but also by the patients and their family members to clearly understand their situation in a ready‐to‐use manner.

Using nomograms to predict prognosis is still emergent in the case of BMC, on which studies were mostly performed in the recent 5 years.^10^, ^11^, ^18^, ^19^ In comparison with models developed in those previous studies, our models may have some inherent advantages. First, we developed models for predicting both CSS and OS with a much larger sample size compared to previous studies,^10^, ^11^, ^18^ which could have significantly improved the power of prediction. Second, we have identified and validated new prognostic factors, for example, primary tumor number and living area, that were not covered before by previous studies. Lastly, the models were thoroughly assessed and validated in the present study. The prediction models were first internally validated by bootstrap resampling from the training cohort. The ROC and calibration curves showed high accuracy and concordance within the prediction of our models, which are comparable with previously reported predictive nomograms of BMC.^10^, ^11^, ^18^ Furthermore, the reproducibility of our nomogram was further validated by an external validation set. The consistency in predictions between the training and validation cohort suggested that our nomogram can be generalized to other BMC populations. Indeed, further validation using the data from our hospital reproduced the performance of the nomograms in terms of ROC and calibration curves. More importantly, we used DCA to assess the clinical applicability of the model and found that using our model to instruct clinical practices could generate more benefits for the patients. Thus, we demonstrate here that our models can be used to guide clinical decisions concerning BMC care and treatment.

Previous studies have shown that there might be differences in factors that determine the OS and CSS, respectively. For example, M staging was found to impact CSS but not OS of BMC patients,^10^ while marital status was found to impact OS only.^18^ Osazuwa‐Peters et al. summarized that the main reasons for being married are to have better clinical outcomes related to health supervision, economic status, support from family, and transportation provided by spouses.^24^ And Wang et al. thought this might explain why married patients may be less likely to die from comorbidities.^18^ In our models, however, M staging and marital status were found to impact both OS and CSS. Given the significant roles played by M staging in determining prognosis, we have the confidence to believe that M staging could impact both OS and CSS. This may, in part, support the power of our models. To our surprise, the number of primary tumors and chemotherapy were only associated with OS but not CSS. We hypothesize that patients with one primary tumor or patients who have undergone chemotherapy are not prone to die from other illnesses. Future studies are warranted to confirm our hypothesis.

Our model has certain limitations. The first limitation roots in the nature of a retrospective study, in which some biases are inevitable. Second, there is a lack of important covariates in the SEER database. For example, due to the time of data collection being prior to the release of the AJCC 8th Edition Cancer Staging Manual, the SEER data do not contain information on lymphovascular invasion or perineural invasion of tumors. Furthermore, treatment options may be coded ambiguously in the SEER database, such as in the case of chemotherapy, where no/unknown chemotherapy treatments are grouped together. Moreover, it has been reported that the histological reports in SEER data may not have been consistently recorded amongst institutions.^25^ Lastly, there might be a wide variation in oncological surgical techniques across multicenter, which are not accounted for within the database.

## 5 CONCLUSIONS

In summary, the nomograms developed in the current study are of great performance in predicting 1‐, 3‐, and 5‐year OS and CSS of BMC patients. Use of the nomograms in clinical practices shall bring significant benefits to BMC patients.

## AUTHOR CONTRIBUTIONS

**Yongmei Tan:** Writing – original draft (lead); writing – review and editing (lead). **Guoxing Huang:** Resources (equal). **Jintao Hu:** Conceptualization (lead); data curation (lead); formal analysis (lead); investigation (lead); methodology (lead); resources (lead); software (lead); validation (lead); visualization (lead). **Shaoping Zhao:** Resources (equal); visualization (equal). **Yanyan Li:** Resources (equal). **Zhihui Wen:** Resources (equal); software (equal). **Liansheng Wang:** Methodology (equal); project administration (equal); resources (equal). **Suling Chen:** Data curation (equal). **Rongxi Chen:** Resources (equal); software (equal). **Haotian Cao:** Validation (equal). **Jinsong Li:** Conceptualization (equal); data curation (equal); formal analysis (equal).

## CONFLICT OF INTEREST STATEMENT

The authors declare no potential conflicts of interest.

## Data Availability

Data sharing is not applicable to this article as no new data were created or analyzed in this study.
